# Ozone-mediated breakdown of microplastics in aqueous environments

**DOI:** 10.1039/d5ja00226e

**Published:** 2025-09-02

**Authors:** Markus A. B. Wieland, Sebastian P. Schwaminger, Matthias Elinkmann, Paul M. Stüger, Jörg Feldmann, David Clases, Raquel Gonzalez de Vega

**Affiliations:** a NanoMicroLab, Institute of Chemistry, University of Graz Graz Austria; b Institute of Inorganic and Analytical Chemistry, University of Münster Münster Germany; c NanoLab, Division of Medicinal Chemistry, Otto Loewi Research Center, Medical University of Graz Graz Austria; d BioTechMed-Graz Graz Austria; e TESLA – Analytical Chemistry, Institute of Chemistry, University of Graz Graz Austria Raquel.gonzalez-de-vega@uni-graz.at

## Abstract

Advanced oxidation processes (AOPs) are increasingly adopted in wastewater treatment to degrade persistent pollutants, including emerging targets such as microplastics (MPs). These particles enter aquatic systems through the fragmentation of bulk plastics and, as their size decreases, exhibit enhanced mobility, surface reactivity, and biological uptake potential. However, the efficiency of AOPs in removing MPs and their nanoscale derivatives (nanoplastics, NPs) remains poorly understood, partly due to the lack of suitable analytical tools. Small MPs and NPs often occur at trace levels and are obscured by colloidal and dissolved background in complex matrices. Moreover, growing evidence suggests that AOPs may promote fragmentation rather than complete degradation. Thus, the focus of this study is to investigate ozone as a reactive agent for MP degradation, using single-particle inductively coupled plasma – mass spectrometry (SP ICP-MS). The formation of nanoscale plastics was qualitatively assessed using dynamic light scattering (DLS). The degradation behaviour of primary MPs such as polystyrene (PS) and polytetrafluoroethylene (PTFE), and secondary MPs generated from bulk poly(methyl methacrylate) (PMMA) and polyvinyl chloride (PVC) was assessed. Ozone exposure led to progressive mass reduction for PS and PMMA, while PTFE and PVC showed greater oxidation resistance. SP ICP-MS revealed detailed transformations in mass, which were projected into size distributions, while DLS confirmed the formation of nanoscale particles in all cases. These findings highlight that ozone-based AOPs can promote nanoplastic formation, underscoring the need to evaluate treatment efficiency not only by particle removal but also with regard to the nature and behaviour of transformation products. The combined use of SP ICP-MS and DLS offers unique insights into MP degradation and the unintended formation of NPs during oxidative treatment, an aspect of particular relevance as AOPs are increasingly integrated into wastewater treatment under the revised European Urban Wastewater Treatment Directive (2024/3019).

## Abstract 2

Ozone-induced degradation of MPs was assessed by single-particle ICPMS, enabling size-resolved detection of polymer-specific fragmentation: PS and PMMA showing pronounced erosion, while PTFE and PVC remained largely unaffected by oxidative breakdown.

## Graphical abstract

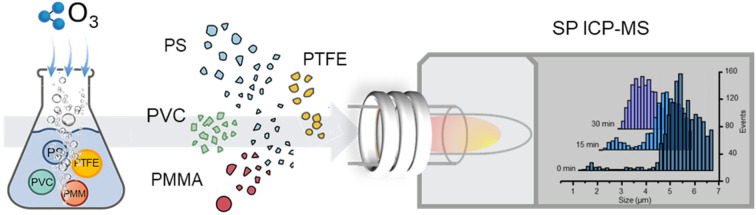

## Introduction

Plastics are indispensable materials in modern society due to their versatility, durability, and low production cost. However, their widespread use and persistence have led to the accumulation of plastic debris in terrestrial and aquatic environments. Over time, bulk plastic undergoes environmental weathering, mechanical abrasion, or chemical transformation, resulting in the formation of microplastics (MPs), particles typically defined as ranging from 1 μm to 5 mm in size.^1^ Continued degradation may produce nanoplastics (NPs, <1 μm),^2^ which are even more mobile, potentially more bioavailable and increasingly difficult to detect. Common pollutant MP polymers include polyethylene (PE), polypropylene (PP), polystyrene (PS), poly(methyl methacrylate) (PMMA), polyvinyl chloride (PVC), and polytetrafluoroethylene (PTFE). These particles can persist in aquatic ecosystems for extended periods, exhibiting high mobility and raising growing concerns about bioaccumulation and long-term environmental and health impacts.^3^

One significant source for MPs in the environment is wastewater treatment plants (WWTP), where MP-containing wastewater is collected and processed. Despite multiple treatment stages, including primary sedimentation, secondary biological degradation, and tertiary disinfection, a substantial fraction of MPs, particularly those in the lower micrometre or nanometre range, escape retention and are discharged into receiving waters. Additionally, MPs may form *in situ* within WWTPs as a result of mechanical stress or chemical exposure.^4,5^ In response to evolving regulatory frameworks, particularly within the European Union, advanced treatment technologies are increasingly being suggested and may become mandatory to mitigate micropollutant emissions from WWTPs.^6–8^ Among these, advanced oxidation processes (AOPs), including ozonation, UV photolysis, and Fenton chemistry, are gaining attention for their potential to degrade MPs.^9^ Ozonation, in particular, is of growing interest, as it can act *via* direct oxidation by molecular ozone (O_3_) as well as indirectly *via* the formation of secondary reactive oxygen species (ROS), such as hydroxyl radicals (˙OH).^10^ These oxidative species can modify polymer surfaces, cleave polymer chains, and induce fragmentation, potentially reducing particle size and modifying reactivity.^11^

However, emerging evidence challenges the assumption that AOPs lead to complete degradation of MPs. Instead, these treatments often induce partial oxidation, embrittlement, and the formation of smaller secondary particles, including nanoplastics.^12^ These transformation products frequently exhibit altered physicochemical properties, such as increased surface reactivity and mobility, which may aggravate their environmental and toxicological impact. In particular, oxidative modification can introduce polar functional groups on MPs' surfaces, enhancing their ability to adsorb co-contaminants such as heavy metals and hydrophobic organic compounds, a phenomenon widely known as the “Trojan Horse” effect.^13–16^ These transformation pathways complicate risk assessments and environmental fate modelling, especially as smaller particles often fall below the detection limit of conventional analytical methods.

Currently, no standardised methods exist to reliably assess the fate of MPs during AOPs, making it difficult to determine whether such treatments effectively degrade, fragment, or simply transform plastic particles. Commonly used analytical methods, including Fourier-transform infrared spectroscopy (FTIR), Raman spectroscopy, and electron microscopy, suffer from limitations in size resolution, matrix compatibility, and particle throughput. As a result, sub-10 μm particles, including those generated through AOP degradation, are often underestimated or entirely overlooked. This situation is aggravated by the relatively low concentrations of MPs in treated effluents and the difficulty of distinguishing polymers from natural colloidal backgrounds. Recent advancements in elemental mass spectrometry, particularly single particle inductively coupled plasmamass spectrometry (SP ICP-MS), offer new opportunities for the detection and characterisation of MPs in complex environmental samples.^17,18^ SP ICP-MS enables high-throughput determination of particle size and number concentrations at environmentally relevant levels, with particular strength in detecting particles in the low-micrometre range (∼1–10 μm), a critical size window that remains challenging to access with conventional optical microscopy and vibrational spectroscopy techniques. In addition to size characterisation, polymer identification may be inferred *via* heteroatom detection^19^ (*e.g.* Cl in PVC or F in PTFE) or through elemental doping, enabling the selective detection of polymer particles in complex matrices.^20,21^ We have previously demonstrated the application of SP ICP-MS for the characterisation of MPs generated from bulk polymers through distinct aging routes, including UV-induced photolysis of PTFE^22^ and ultrasonication of PS and PVC, showing that degradation conditions strongly influence particle morphology, oxidation state, and size distribution.

The present study investigates the potential of SP ICP-MS for monitoring microplastics degradation during AOPs, with a focus on particle mass reduction and the formation of secondary MPs. Complementary DLS was employed to demonstrate the formation of nanoscale particles and evaluate whether complete particle elimination occurs. The combined use of SP ICP-MS and DLS offers a powerful analytical framework to guide the implementation and optimisation of AOPs while also identifying operational conditions that may inadvertently promote particle shrinking and/or fragmentation rather than removal.

## Experimental

### Chemicals and consumables

PS (5.09 ± 0.07 μm, aqueous suspension, 10% solids) and europium-labelled PS microparticles (6.3 ± 0.1 μm, aqueous suspension, 2.5% solids) were purchased from microParticles GmbH (Berlin, Germany). Additionally PS particles (10 μm, aqueous suspension, 10% solids) were obtained from Sigma Aldrich. All aqueous suspension were stored at 4 °C. Solid PTFE Particles with a mean particle size of 3 μm were purchased from Polysciences (Warrington, PA, USA). PMMA (PLEXIGLAS® glass sheet, transparent, non-reflecting 1.50 × 120 × 250 mm) and PVC (Rigid-PVC, transparent, colorless 0.5 × 297 × 420 mm, A3) bulk plastic sheets were bought from Modulor (Berlin, Germany). Elemental Cl and F standards at a concentration of 1000 mg L^−1^ (Single-Element ICP Standard-Solution Roti®Star, Carl Roth®, Karlsruhe, Germany), a Ba solution prepared from barium nitrate (Ba(NO_3_)_2_ ≥99%, ACS, Merck) and a C solution prepared from sodium carbonate (Na_2_CO_3_ ≥99,5%, p.a., ACS, anhydrous, Carl Roth®), were diluted to working concentrations using ultrapure water (18.2 MΩ cm, Merck Millipore, Bedford, USA). The concentrations used for each polymer type during the experiments are provided in Table S1, all MPs suspensions were stored in glass vials.

### Ozone generator

**Fig. 1:**
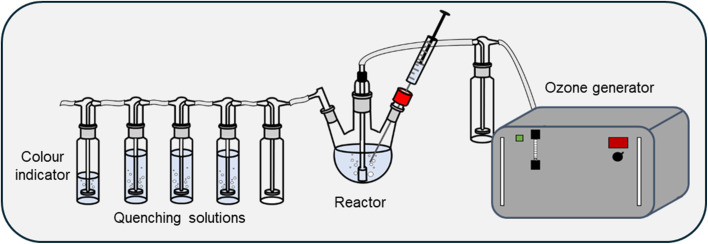
Experimental setup for the degradation of microplastics using ozone.

The inlet of a COM-AD-04 ozone generator (Anseros, Tübingen, Germany) was connected to a high-purity oxygen cylinder (99.999%, Messer Austria GmbH). The ozone-rich output stream was first directed into a safety flask and subsequently passed through a gas dispersion frit, which was securely inserted into a three-necked round-bottom flask. To ensure an airtight seal, the joint between the frit and the flask was wrapped with parafilm. A septum was installed on the second neck to enable sampling during operation. The third neck was connected in series to a cascade of five gas wash bottles. The first bottle remained empty, serving as a buffer zone or moisture trap. The following three bottles were each equipped with a frit and filled with 200 mL of an aqueous sodium sulphite solution (Na_2_SO_3_·7H_2_O, 50 g L^−1^), acting as an efficient ozone quenching system. The final wash bottle contained 100 mL of an aqueous potassium iodide solution (KI, 5 g L^−1^), serving as a qualitative indicator for unquenched ozone by visual detection of iodine formation, thus confirming the exhaustion of the quenching capacity. All glass joints were carefully silicon greased and secured using suitable clamps to maintain the integrity of the system under continuous gas flow. The ozone generator was operated at full capacity (100% output) during all experimental procedures to ensure reproducibility and controlled ozone delivery. Blank runs using ultrapure water were performed to assess the presence of C-containing particles. These samples were subjected to the optimised ozone treatment procedure, with aliquots collected and analysed by SP ICP-MS. No formation of carbon-based particles was observed as a result of ozone exposure or interactions with components of the reactor system (see Fig. S1). To further evaluate the potential presence of nanoscale particles, the same blank samples were analysed by DLS. No measurable scattering signal was detected, indicating the absence of detectable nanoparticles within the sensitivity range of the technique. The experimental set up is shown in Fig. 1.

After the ozone exposure step, aliquots were transferred into open glass vials and left uncapped at room temperature for several minutes. This allowed residual ozone to dissipate, as ozone is unstable in aqueous media and rapidly decomposes or volatilises upon air exposure. Although no chemical quenching was applied, the open handling conditions and the known short half-life of ozone in water ensure that further ozone-driven reactions after sampling were minimised. All samples were analysed on the same day as the experiment. In addition, storage stability tests under different conditions (room temperature and refrigeration) and time points were performed; the results are provided in the SI (Fig. S2).

### Milling of bulk plastics

The PMMA and PVC bulk plastic sheets were first cut into smaller pieces (approximately 3 × 3 mm) using the in-house workshop facilities. These pieces were then cryogenically cooled with liquid nitrogen and subjected to two stages of milling using a ZM 1000 centrifugal mill (Retsch, Haan, Germany). The initial milling was performed with a 4 mm sieve, followed by a second milling with a 1 mm sieve, both at a rotational speed of 10 000 rpm. To further reduce the particle size, the shredded plastic was subsequently milled for 2 hours using a ball mill. The resulting plastic powder was then dispersed in ultrapure water, and subsequently passed through a paper filter (15–20 μm pore size) to remove larger particles and agglomerates. This filtration step was crucial to prevent clogging of tubing and nebuliser during SP ICP-MS analysis.

### Single particle ICP-MS

SP detection was performed using an 8900 series ICP-MS system (Agilent Technologies, Santa Clara, CA, USA). The system was equipped with a Scott-type double-pass spray chamber maintained at 2 °C, and a MicroMist™ concentric nebuliser (Elemental Scientifc Inc., Omaha, NE, US) was used for sample nebulisation. The instrument was operated in MS/MS mode and the dwell time of the quadrupole was set to 100 μs. The RF power was set to 1.6 kW, the sample depth (*z*-position) was set to 9 mm with a nebuliser gas flow of 1.3 L min^−1^, and hydrogen (0.5 mL min^−1^) was introduced in the collision/reaction cell (CRC) to enhance the signal to noise ratio.

Data acquisition was performed using the MassHunter software (Agilent Technologies) and subsequent data analysis was conducted using the open-source python-based data processing platform “SPCal”.^23,24^ For event discrimination, an iterative Gaussian thresholding method was employed with a *z*-value (*σ*) of 8 ensuring high sensitivity and specificity in particle detection. For polymers particles composed exclusively of C, particle size determination was performed using the ^13^C and evaluated *via* the “mass response” method, employing 5 μm PS-MPs as reference particle. The observable size window was estimated between 1 and 10 μm, with a size detection limit (sDL) of 0.8 μm based on ^13^C detection.

Size calibrations for both PVC and PTFE particles were performed using the particle mass-based transport efficiency approach.^25^ For PVC, the ^35^Cl signal was monitored, while PTFE particles were evaluated *via* the formation of the ^138^Ba^19^F molecular ion. Ionic response factors for F, Cl and C were established using a series of dissolved standards. Transport efficiency, estimated at approximately 6%, was calculated based on the C ionic signal and a 5 μm polystyrene (PS) standard. The calibration protocol for PTFE followed the method described by Gonzalez de Vega *et al.*,^22^ with the modification that 10 mg L^−1^ of Ba solution were added directly to the sample suspensions, rather than introduced *via* a T-piece.

### Dynamic light scattering

A 5 μm polystyrene (PS) particle standard was used to assess the performance of dynamic light scattering (DLS) measurements on degraded samples obtained from the ozonation experiments. Hydrodynamic diameters were analysed using a VASCO Flex instrument (Cordouan Technologies SAS, France). Typical sample volumes were approximately 2 mL and loaded into a cuvette. Each sample was analysed in triplicate, with an acquisition time of 120 seconds per run, at pH 7.0 and 25 °C. Refractive index (RI) of 1.59 was used for the calculations.

## Results and discussion

### Evaluation of ozone reactor flow conditions

**Fig. 2:**
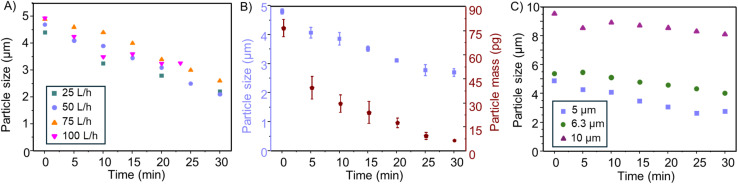
(A) Optimisation of ozone flow rates using a 5 μm PS standard, (B) assessment of degradation efficiency and method reproducibility based on particle size and mass using a 5 μm PS standard (*n* = 3), (C) evaluation of ozone's effect on particle size across 5, 6.3 and 10 μm PS particles.

Optimisation of the ozone reactor was conducted by systematically varying the oxygen input flow rates and evaluating their effect on particle degradation. A suspension of 5 μm PS microplastics was selected as a model system. Aliquots were collected at 5-minute intervals over a 30-minute period, and particle size distributions were analysed *via* SP ICP-MS. The calibrated particle sizes corresponding to different contact times and flow rates are illustrated in Fig. 2A. Across all tested flow rates (25, 50, 75 and 100 L h^−1^), a consistent reduction in particle mass was observed. This reduction is hypothesised to be linked into a reduction in size. Assuming spherical diameter and surface erosion, a reduction of approximately 1.5 μm, was observed after 20 minutes of ozone exposure. No significant trend indicating enhance degradation at higher flow rates was observed, suggesting that beyond a certain threshold, increasing oxygen flow did not further improve degradation efficiency. The absence of a flow-dependent effect on degradation was likely related to ozone saturation in water. At 20 °C, ozone exhibits a solubility of approximately 570 mg L^−1^, which appeared to be reached under all tested flow rates.^26^ Although 25 L h^−1^ was sufficient to achieve saturation, it proved difficult to regulate and maintain a stable output with the ozone generator, resulting in inconsistent flow and limiting reliable sampling. At the highest tested flow rate (100 L h^−1^), Na_2_SO_3_ quenching capacity was exhausted after 23 minutes, and the experiment was terminated at this point. Based on these operational considerations and to ensure stable and reproducible ozonation, a flow rate of 50 L h^−1^ was selected for all subsequent experiments.

To ensure the reliability of the ozonation procedure, triplicate experiments were performed using identical suspensions of 5 μm PS standard particles. Each suspension was subjected to 30 minutes of ozone exposure, with aliquots collected at 5-minute intervals and analysed by SP ICP-MS. Particle size and mass distributions were generated for each time point, and mean values were compared across the three experiments. As shown in Fig. 2B, the degradation profiles were highly consistent and relative standard deviations (RSDs) between experiments remained below 8% at all time points, confirming the repeatability of the ozonation procedure under the selected conditions.

MPs degradation under oxidative conditions is proposed to follow two principal, and potentially co-occurring, pathways:^27,28^ (1) gradual mass loss which may be caused by surface erosion of individual particles, and (2) fragmentation, in which particles break apart into smaller discrete fragments as a result of structural weakening or localised oxidative degradation. SP ICP-MS is a mass-sensitive method, and it is possible that both surface reduction and increasing porosity led to C mass loss in single particles. Single particles masses detected in SP ICP-MS can be calibrated into sizes using polymer density and C-mass fraction. Assuming that particle density is not changed during oxidation, size reduction can be visualised and is discussed in the following. This assumption is consistent with the established ozonation chemistry of PS, where ozone primary attacks the aliphatic backbone and benzylic positions at the accessible particle surface, through oxidative pathways, resulting in chain scission and the formation of oxygen-containing functional groups.^29^ Consequently, we suggest that PS particles undergo surface erosion, characterised by a measurable decreased in particle size and particle mass.^30^ Although fragmentation was not directly evident in the SP ICP-MS size distributions, it cannot be excluded, particularly at advanced stages of degradation or in more oxidation-prone polymers. The potential release of smaller nanoscale plastic fragments was further examined using dynamic light scattering (DLS) as described in the following section.

**Fig. 3:**
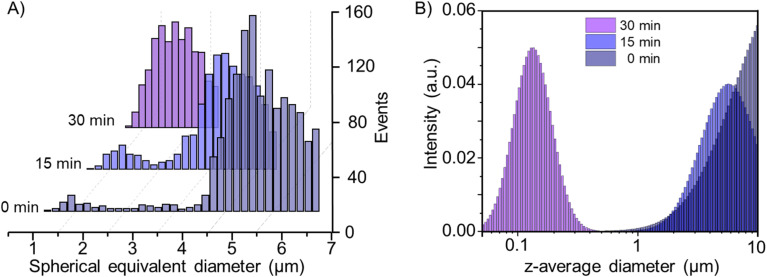
Size distribution histograms of a 5 μm PS standard after exposure to ozone for 0, 15 and 30 min, obtained by (A) SP ICP-MS and (B) DLS intensity distribution analysis.

A more detailed size analysis of MPs is shown in Fig. 3A, illustrating the continuous breakdown of the PS standard under ozone exposure over time (0, 15 and 30 min). At 0 min, the size histogram displays a dominant population centred around 5 μm, representing the intact starting material with two minor fractions at approximately 3.5 μm and 1.5 μm. After 15 min of ozone exposure, the predominant particle size shifts to around 3.5 μm, with a slight increase in the fraction at 1.5 μm, suggesting partial surface degradation and fragmentation of the original particles. Finally, after 30 min, the distribution is centred around an average size of 2 μm, suggesting that continued oxidation further breaks down the particles into smaller fragments. In addition to the reduction in particle mass and size, a notable decline in the number of detectable particles was observed over time. This suggests that, beyond gradual surface erosion, a subset of the particles may have undergone complete degradation or transformation into fragments smaller than the SP ICP-MS detection limit (∼1 μm). Concurrently, a marked increase in the continuous carbon background signal was recorded, consistent with the release of dissolved or colloidal degradation products such as oxidized oligomers or low-molecular-weight fragments. This effect is particularly evident for the 5 μm PS standard, as shown in Fig. S4, where the background carbon signal increases with ozone exposure. Together, these findings indicate that ozone exposure not only reduces particle mass but also contributes to the generation of non-detectable transformation products.

This progression highlights a dynamic degradation process, where particle erosion, decreasing counts, and elevated background signals coincide with the emergence of intermediate size fractions, ultimately supporting the formation of smaller MPs and potentially NPs through fragmentation and ozone-mediated erosion.

### Formation of secondary MPs and NPs resulted from O_3_ degradation

To further validate the particle size evolution observed by SP ICP-MS, dynamic light scattering was employed to analyse the hydrodynamic diameter of PS particles subjected to ozonation. While the MP standard was initially considered relatively monodisperse, at 0 min, SP ICP-MS analysis of the 5 μm PS standard revealed two smaller particle populations (compare Fig. 3A). This finding was corroborated by DLS, which reported a *z*-average diameter of 4.4 μm and exhibited a broadened size distribution (Fig. 3B). The corresponding polydispersity index (PDI) at 0 min was 0.5565, indicating a highly polydisperse system. These effects, together with the intensity-weighted detection principle of DLS and its limited ability to resolve complex, polydisperse systems, especially near its upper detection limit of ∼10 μm, pose known challenges for accurate particle sizing in heterogeneous samples. It is worth pointing out that DLS analysis of a polydisperse system can be challenging and artefacts through aggregates and irregular shapes are possible. In context of the study, DLS was used to confirm the formation of smaller and specifically nanoscale plastics and absolute values need to be interpreted carefully.

Despite these constraints, DLS effectively captured the overall trend in particle degradation. After 15 minutes of ozonation, the hydrodynamic diameter decreased to 3.7 μm, with a PDI of 0.3354, reflecting a moderately polydisperse system. Although still broad, the decrease in PDI suggests a shift towards a more uniform particle population as larger aggregates began to break down, which is in close agreement with the 3.5 μm mean particle size determined by SP ICP-MS. Unlike SP ICP-MS, DLS was not able to resolve the underlying size distribution or distinguish sub-populations, instead presenting a single, averaged peak, underscoring the superior population-resolving power of SP ICP-MS in the micrometre range. Following 30 minutes of ozone exposure, DLS revealed a shift in the size distribution, with the emergence of a predominant population centred around 100 nm, strongly indicating the formation of nanoplastics. The associated PDI dropped further to 0.1463, suggesting a relative narrow size window distribution of the newly formed nanoparticles. These particles fall below the size detection limit of SP ICP-MS (∼1 μm). Notably, no measurable scattering signal was detected in the ozone-treated blanks, confirming that the observed nanoscale particles originate from polymer degradation rather than reactor artefacts or background colloids. The appearance of sub-micrometre particles highlights the complementarity of the two techniques: SP ICP-MS enables detailed, element-specific, particle-resolved analysis of MPs populations, while DLS extends detection into the nanometre range, capturing transformation products beyond the size detection limit of mass-based single-particle methods.

Together, these results demonstrate that ozone-induced oxidative degradation promotes a continuous breakdown of PS particles, yielding both secondary MPs and NPs. The integration of SP ICP-MS and DLS thus provides a robust, multidimensional approach for characterising degradation pathways across a broad size spectrum. Such approach is essential for advancing our understanding of the fate and transformation of polymer-derived particles in environmental and biological systems, particularly in the context of wastewater treatment plant using ozonation as the fourth treatment stage.

To explore whether nanoplastic formation is polymer-specific, additional DLS measurements were performed on ozone-treated suspensions of PTFE, PMMA, and PVC MPs. In all three cases, a distinct shift in size distribution towards the nanoscale was observed following ozone exposure, supporting the formation of nanoplastics, which were beyond the detection limit of SP ICP-MS. These findings indicate that ozone-induced transformation to nanoscale particles is a generalised phenomenon across chemically diverse polymers. The corresponding DLS intensity distributions are provided in Fig. S3, and SP ICP-MS size degradation profiles for these polymers are discussed in the subsequent sections.

### Impact of particle size on degradation efficiency

A wide range of MPs sizes are to be expected in wastewater treatment systems and environmental matrices.^31^ To systematically investigate size-dependent degradation behaviour, PS particles of varying diameters were subjected to controlled ozonation, and their size reduction monitored at defined time points using SP ICP-MS. This method enabled rapid detection of particle degradation within the 1–10 μm size range. However, the analysis of particles smaller than 1 μm was hindered by increasing background signals arising from the degradation byproducts. This effect is attributed to the accumulation of oxidised low-molecular-weight fragments, colloidal debris, and/or dissolved organic carbon released during progressive surface erosion. These degradation products contribute to an elevated continuous carbon signal, which was found to intensify with decreasing particle size and prolonged ozone exposure (see Fig. S4). The resulting increase in baseline can obscure particle events and compromise the accuracy of size determination near the lower detection limit, particularly for particles approaching the submicron range. In contrast, larger particle are subject to a reduced transport efficiency through the spray chamber and particles exceeding 10 μm were hardly transmitted into the plasma, restricting their detection.^32^ Therefore, PS particles of 5, 6.3 and 10 μm were selected for experimental evaluation, and mean particles sizes obtained during and after degradation are presented in Fig. 2C. All tested particle sizes exhibited a clear reduction in diameter over time. Specifically, 5 μm particles decreased by 2 μm, while both the 6.3 μm and 10 μm particles were reduced by approximately 1.5 μm. This degradation pattern observed under ozone exposure supports a surface-limited oxidative mechanism, where smaller particles exhibit more rapid size reduction due to their higher surface area-to-volume ratio.^33^ However additional replicates and sizes are required to validate this trend. Notably, these results demonstrate that PS MPs are susceptible to ozone-induced degradation across the tested size range, underscoring the general applicability of the methods and its potential relevance for diverse environmental plastic pollutants.

### Influence of ozone degradation on polymer type

The degradation behaviour of MPs exposed to ozone is greatly influenced by the chemical composition of the polymer.^34^ Different polymer types exhibit varying susceptibility to oxidative degradation, determined by their molecular structure, bond stability, and surface reactivity.^35^ In this study, we systematically evaluated the influence of ozone degradation on two distinct polymer types: polystyrene (PS) and polytetrafluoroethylene (PTFE). These polymers were chosen for their contrasting chemical characteristics, PS, an aromatic hydrocarbon polymer, is known for its moderate oxidative resistance, while PTFE, a perfluorinated polymer, is recognised for its exceptional chemical inertness.

**Fig. 4:**
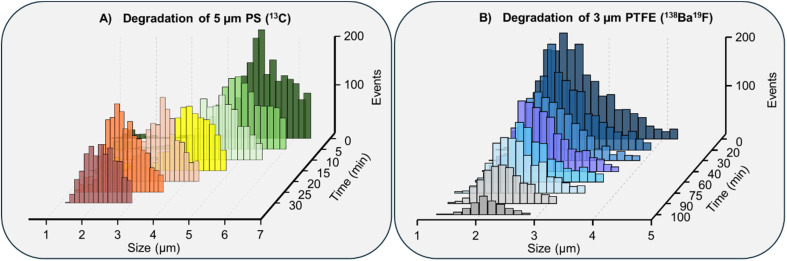
Influence of ozone degradation on different polymer types. Calibrated size histograms of detectable MPs from: (A) a 5 μm PS standard targeting ^13^C signal, and (B) a 3 μm PTFE standard targeting ^138^Ba^19^F signal.

By exposing these polymers to controlled ozone treatment, we aimed to characterise the degradation dynamics of each polymer type, examining the extent of particle size reduction, the formation of smaller fragments, and changes in detectable elemental signals. The calibrated size distributions of the resulting particles, targeting characteristic elemental signals (^13^C for PS and ^138^Ba^19^F for PTFE) are illustrated in Fig. 4. For PS, ozone exposure resulted in a pronounced reduction in particle size (Fig. 4A). SP ICP-MS analysis revealed a substantial decrease in the number of intact 5 μm particles, accompanied by the emergence of smaller fragments, indicative of secondary MPs. While SP ICP-MS cannot directly confirm the formation of nanoparticles, complementary DLS measurements revealed a nanoscale population, supporting the hypothesis that oxidative degradation also produces nanoplastics under these conditions. Calibrated size distributions further confirmed these transformations, showing a clear shift in average particle from 5 μm to approximately 2 μm over 30 min of ozone exposure.

In contrast, PTFE demonstrated high resistance to ozone-induce degradation. Even under extended exposure conditions, up to 100 minutes only a size reduction of around 0.5 μm was detected (Fig. 4B). This exceptional stability is attributed to the strength of the carbon–fluorine (C–F) bond, which has a bond dissociation energy of around 485 kJ mol^−1^, significantly higher than that of a typical carbon, carbon (C–C) bond (∼350 kJ mol^−1^). The high C–F bond energy confers exceptional chemical resistance under oxidative stress, making PTFE remarkably stable even in highly reactive environments.^36^ Despite the limited size reduction, a gradual decline in the number of detectable particles was observed (Fig. 4B), accompanied by an increase in the continuous carbon background signal (Fig. S5). This suggests that ozone exposure may still lead to partial degradation or transformation of PTFE particles, resulting in the release of low-molecular-weight or colloidal species below the detection threshold of SP ICP-MS. The obtained size distributions were further corroborated by monitoring the ^13^C signal, providing an independent confirmation of the observed trend; a detailed comparison is presented in Fig. S5. This comparative analysis highlights that ozone, as an advanced oxidation process, exhibits polymer-dependent reactivity, emphasising the critical role of polymer chemistry in determining the extent to which MPs are transform into secondary MPs or NPs.

### Degradation of MPs generated from milled plastic debris

**Fig. 5:**
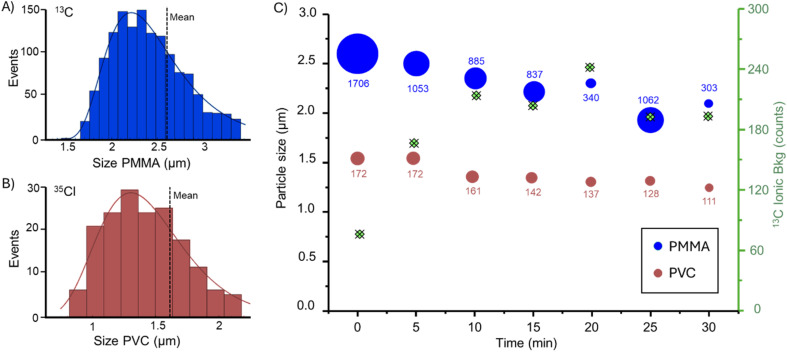
Formation of MPs and size characterisation from bulk plastic after the milling process, specifically, (A) PMMA detected *via*^13^C signal and, (B) PVC detected *via*^35^Cl signal, both by SP ICP-MS. (C) Degradation of milled PMMA and PVC MPs by ozone, represented by a bubble plot that combines particle size and number of detected particles. The corresponding ^13^C background signal for PMMA is also shown for each time point.

The degradation behaviour of different MP types derived from bulk plastic materials was further investigated. The resulting size distributions for the extracted PMMA and PVC MPs are shown in Fig. 5A and B, respectively. SP ICP-MS characterisation revealed that PMMA-derived MPs had an average size of approximately 2.6 μm, based on the ^13^C signal, while PVC-derived MPs averaged around 1.6 μm, based on the ^35^Cl signal, assuming spherical shape. These secondary MPs, produced from real bulk plastic samples were then exposed to ozone for 30 minutes following the previously described protocol. This allowed for assessment of ozone-induced degradation across different polymer types under more realistic conditions. Fig. 5C displays the evolution of particle size and number of detectable particles over time. PMMA MPs exhibited a notable reduction in average size from 2.6 μm to 1.9 μm within 25 minutes of ozone exposure. In contrast, PVC MPs showed only a slight decrease, from 1.6 μm to 1.3 μm, over the same period. These findings highlight the polymer-dependent nature of ozone degradation, with PMMA demonstrating a higher susceptibility to oxidative fragmentation than PVC.

The distinct oxidative degradation behaviours observed between PMMA and PVC, can be attributed to their distinct chemical structures and reactivities toward oxidising agents such as ozone. PMMA is more susceptible to oxidation due to the presence of tertiary carbon atoms and ester groups, promoting the generation and stabilisation of radicals, which leads to chain scission and breakdown.^37^ In contrast, PVC contains chlorine atoms on its backbone, which initially reduce ozone-induced oxidative degradation by suppressing hydrogen abstraction and limiting oxidation to the polymer surface. However, once degradation starts, especially from heat or UV light, PVC breaks down quickly through dehydrochlorination and radical chain reactions.^38^ As a result, PMMA undergoes more extensive and rapid degradation than PVC under the same oxidative conditions, consistent with the known chemical reactivity of these materials and further supports the conclusion that ozone-mediated breakdown is strongly polymer-specific. It is worth noting that the generation of oxidised low-molecular-weight species during polymer degradation could contribute to elevated dissolved organic carbon (DOC) levels in the treated water, an aspect that may represent an unintended consequence of advanced ozonation steps in wastewater treatment.

## Conclusions

This study demonstrates that ozone treatment induces size-dependent and polymer-specific degradation of microplastics, resulting in the formation of smaller fragments, including nanoplastics. Through SP ICP-MS, we studied size reductions and observed particle transformation in both primary and secondary MPs. Under the tested conditions, PS and PMMA exhibited pronounced degradation, while PTFE and PVC showed greater resistance to oxidative breakdown. Complementary analysis by DLS revealed the formation of nanoscale plastic particles in all analysed samples, supporting the hypothesis that oxidative treatment may also lead to nanoplastic formation, and reinforcing the potential of combined analytical approaches to monitor MP fragmentation.

These findings underscore that the implementation of AOPs, while effective in transforming MPs, may shift plastic pollution toward smaller, more mobile, and potentially more bioavailable forms rather than fully eliminating it. As such, the efficacy of AOPs must be evaluated not only in terms of removal efficiency, but also with respect to the nature and environmental behaviour of the transformation products. SP ICP-MS, especially when supported by orthogonal techniques such as DLS, represents a powerful analytical platform to study MP and NP formation under treatment conditions and provides essential insight for the environmental risk assessment of oxidative degradation pathways. In this context, and with the revised European Urban Wastewater Treatment Directive (2024/3019) promoting the use of AOPs for advanced micropollutant removal, our findings highlight the need to consider potential side effects such as nanoplastic formation. These considerations are essential when implementing such technologies in wastewater treatment strategies.

## Conflicts of interest

There are no conflicts to declare.

## Supplementary Material

JA-040-D5JA00226E-s001

## Data Availability

The data supporting this article have been included as part of the SI. See DOI: https://doi.org/10.1039/d5ja00226e.
