# Supplementary material for: Ozone-mediated breakdown of microplastics in aqueous environments
Source: J Anal At Spectrom. 2025 Sep 2;40(10):2870–8. doi: 10.1039/d5ja00226e (PMC12418336; doi:10.1039/d5ja00226e)

Supplementary Information (SI) for Journal of Analytical Atomic Spectrometry.
This journal is © The Royal Society of Chemistry 2025

# Supplementary information

## Ozone-Mediated Breakdown of Microplastics in Aqueous Environments

Markus A. B. Wieland[1,2], Sebastian Schwaminger[3,4], Matthias Elinkmann[1], Paul M. Stüger[5], Jörg Feldmann[5], David Clases[1], Raquel Gonzalez de Vega[5]*

[1]NanoMicroLab, Institute of Chemistry, University of Graz, Graz, Austria

[2]Institute of Inorganic and Analytical Chemistry, University of Münster, Münster, Germany

[3]NanoLab, Division of Medicinal Chemistry, Otto Loewi Research Center, Medical University of Graz, Graz, Austria

[4]BioTechMed-Graz, Graz, Austria

[5]TESLA- Analytical Chemistry, Institute of Chemistry, University of Graz, Graz, Austria

*Corresponding author: Raquel.gonzalez-de-vega@uni-graz.at

## Table of Contents

**Table S1**: Experimental conditions used in the ozonation experiments, including oxygen flow rates, plastic sources, polymer types, particle sizes, initial amounts of microplastic material, and volumes of ultrapure water used for sample preparation.

**Figure S1:** Raw $^{13}C$ signals from SP ICP-MS measurements of a blank sample (ultrapure water) subjected to the same experimental procedure. No $^{13}C$ particle signals were detected at either 0 or 30 minutes, confirming that no carbon-based particles were formed as a result of ozone exposure or interactions with components of the reactor system.

**Figure S2**: Storage stability tests of two 5 μm PS standard solutions after ozone treatment, evaluated under different conditions (room temperature and refrigeration) over multiple time points. The data represent the average particle size at each time point for the respective storage conditions.

**Figure S3**: DLS intensity distributions of PMMA, PVC, and PTFE microparticles before and after ozone treatment, demonstrating the formation of nanoscale plastic fragments. Note: For PMMA and PVC, DLS analyses were performed using a newly prepared batch of milled

particles following the same milling and filtration procedure described in the Experimental section.

**Figure S4**: Ozone-induced degradation of a 5 µm polystyrene (PS) standard over time, represented by a bubble plot combining particle size and the number of detected particles. In addition, the $^{13}C$ background signal is shown for each time point, indicating a progressive increase in dissolved or colloidal carbon species as degradation proceeds.

**Figure S5**: Ozone induce degradation of 3 µm PTFE particles, monitored via $^{138}Ba^{19}F$ and $^{13}C$ signals, represented by a bubble plot that combines particle size and number of detected particles. In addition, the $^{13}C$ background signal is shown for each time point.

**Table S1**: Experimental conditions used in the ozonation experiments, including oxygen flow rates, plastic sources, polymer types, particle sizes, initial amounts of microplastic material, and volumes of ultrapure water used for sample preparation.

| Name of the experiment | Oxygen flow rate (L/h) | Plastic Source | Amount of plastic | | Amount of water (mL) |
|---|---|---|---|---|---|
| | | | (µL) | (mg) | |
| Optimisation of the generator setup 1 | 25 | 5 µm Polystyrene Particles | 40 | - | 100 |
| Optimisation of the generator setup 2 | 50 | 5 µm Polystyrene Particles | 40 | - | 100 |
| Optimisation of the generator setup 3 | 75 | 5 µm Polystyrene Particles | 40 | - | 100 |
| Optimisation of the generator setup 4 | 100 | 5 µm Polystyrene Particles | 40 | - | 100 |
| Reproducibility Test 1 | 50 | 5 µm Polystyrene Particles | 40 | - | 100 |
| Reproducibility Test 2 | 50 | 5 µm Polystyrene Particles | 40 | - | 100 |
| Reproducibility Test 3 | 50 | 5 µm Polystyrene Particles | 40 | - | 100 |
| Degradation of 6.3 µm Eu-particles | 50 | 6.3 µm Polystyrene Particles Eu | 100 | - | 100 |
| Degradation of 10 µm particles | 50 | 10 µm Polystyrene Particles | 160 | - | 80 |
| Degradation of PTFE particles | 50 | PTFE Particles 3000 nm | - | 11 | 100 |
| Degradation of milled PVC particles | 50 | Milled PVC Particles | - | 150 | 80 |
| Degradation of milled PMMA particles | 50 | Milled PMMA Particles | - | 80 | 100 |

**Figure S1:** Raw $^{13}C$ signals from SP ICP-MS measurements of a blank sample (ultrapure water) subjected to the same experimental procedure. No $^{13}C$ particle signals were detected at either 0 or 30 minutes, confirming that no carbon-based particles were formed as a result of ozone exposure or interactions with components of the reactor system.

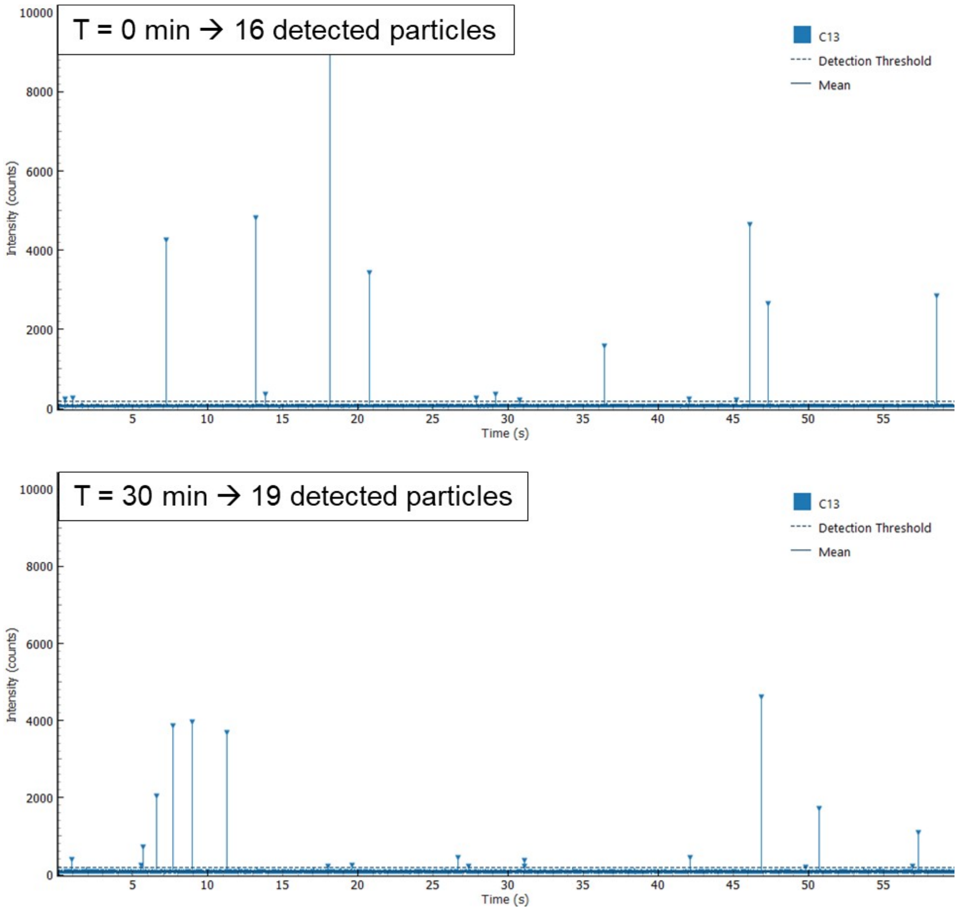

**Figure S2**: Storage stability tests of two 5 μm PS standard solutions after ozone treatment, evaluated under different conditions (room temperature and refrigeration) over multiple time points. The data represent the average particle size at each time point for the respective storage conditions.

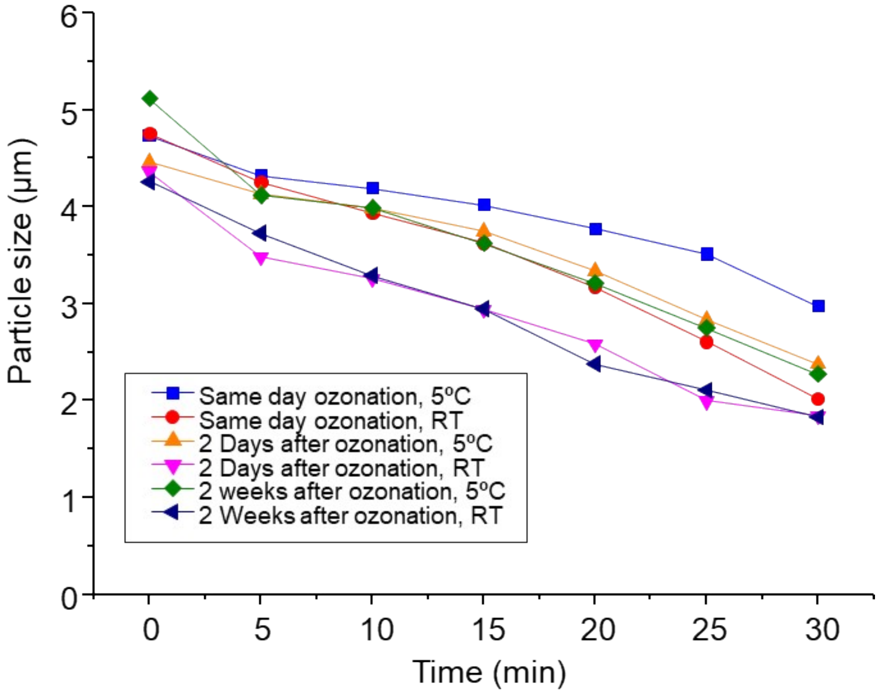

**Figure S3:** DLS intensity distributions of PVC, PMMA and PTFE MPs before and after ozone treatment, demonstrating the formation of nanoscale plastic fragments. Note: For PMMA and PVC, DLS analyses were performed using a newly prepared batch of milled particles following the same milling and filtration procedure described in the Experimental section.

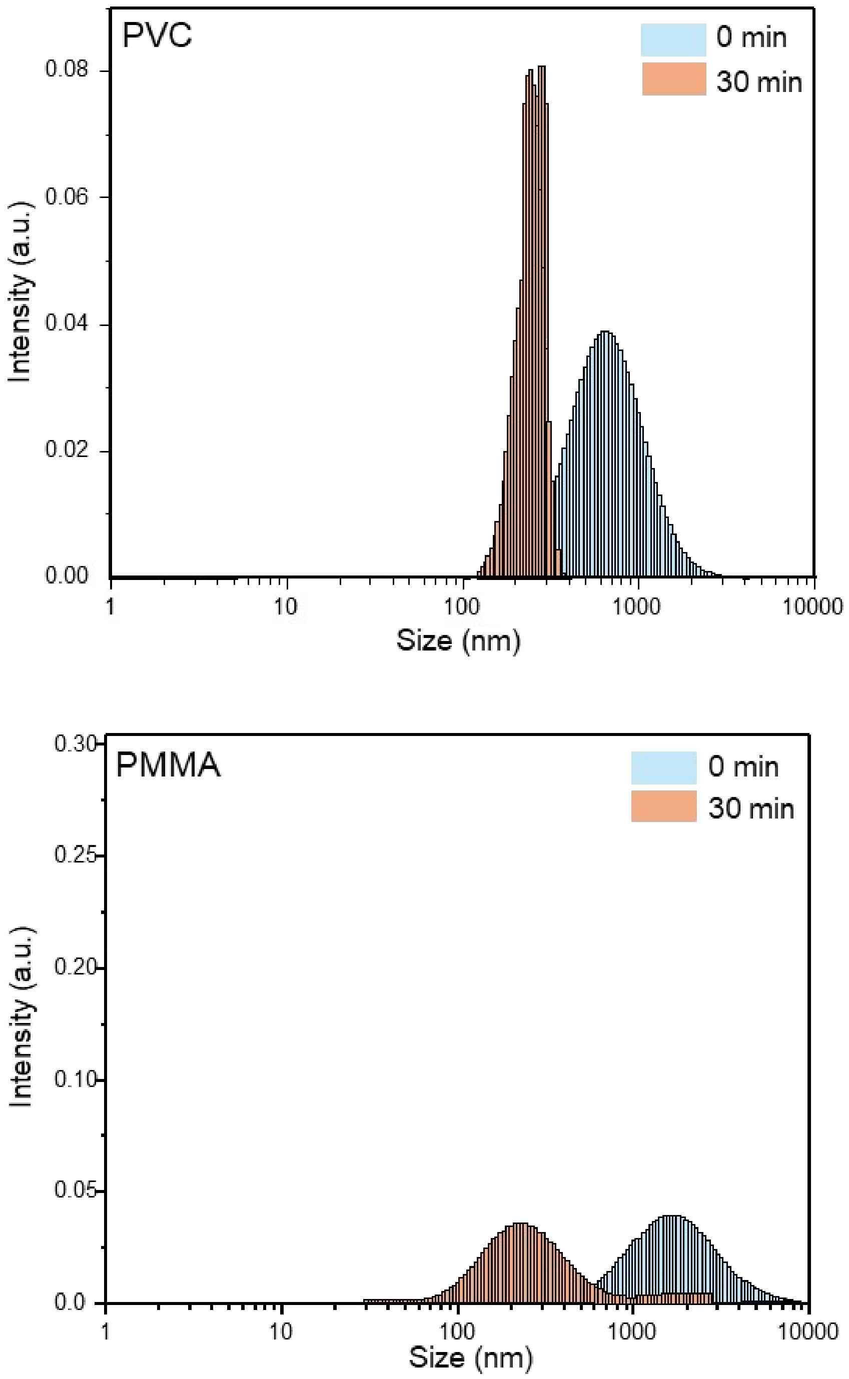

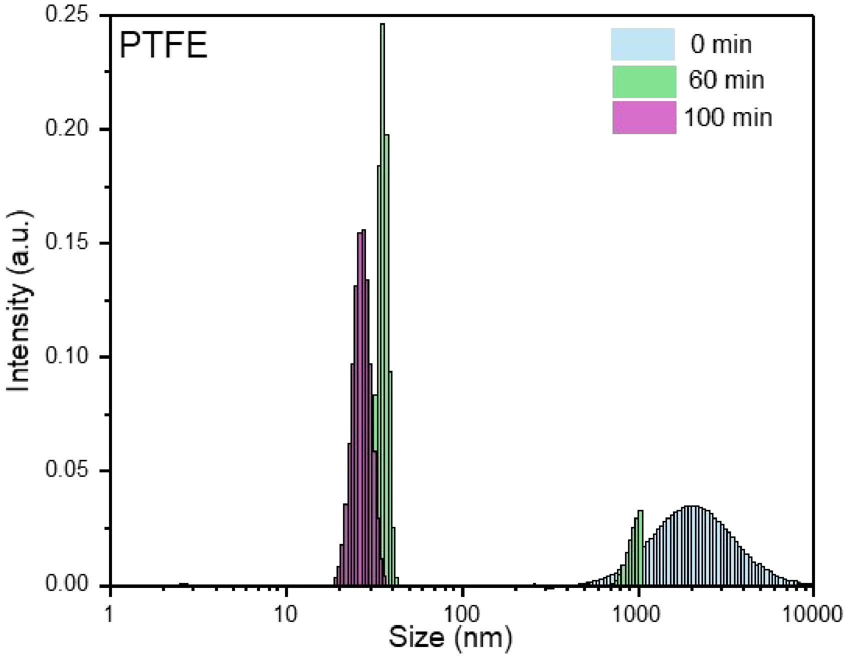
PTFE
0 min
60 min
100 min
Intensity (a.u.)
0.00
0.05
0.10
0.15
0.20
0.25
1
10
100
1000
10000
Size (nm)

**Figure S4:** Ozone-induced degradation of a 5 μm polystyrene (PS) standard over time, represented by a bubble plot combining particle size and the number of detected particles. In addition, the $^{13}C$ background signal is shown for each time point, indicating a progressive increase in dissolved or colloidal carbon species as degradation proceeds.

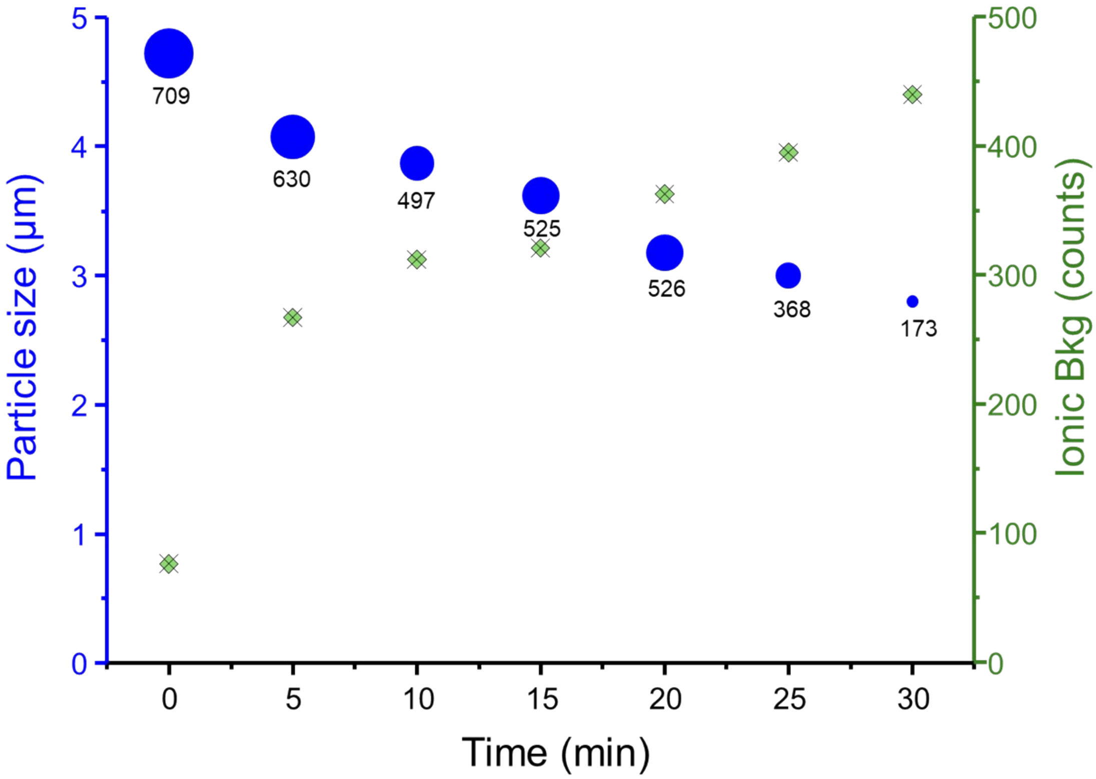

**Figure S5**: Ozone induce degradation of 3 µm PTFE particles, monitored via $^{138}Ba^{19}F$ and $^{13}C$ signals, represented by a bubble plot that combines particle size and number of detected particles. In addition, the $^{13}C$ background signal is shown for each time point.

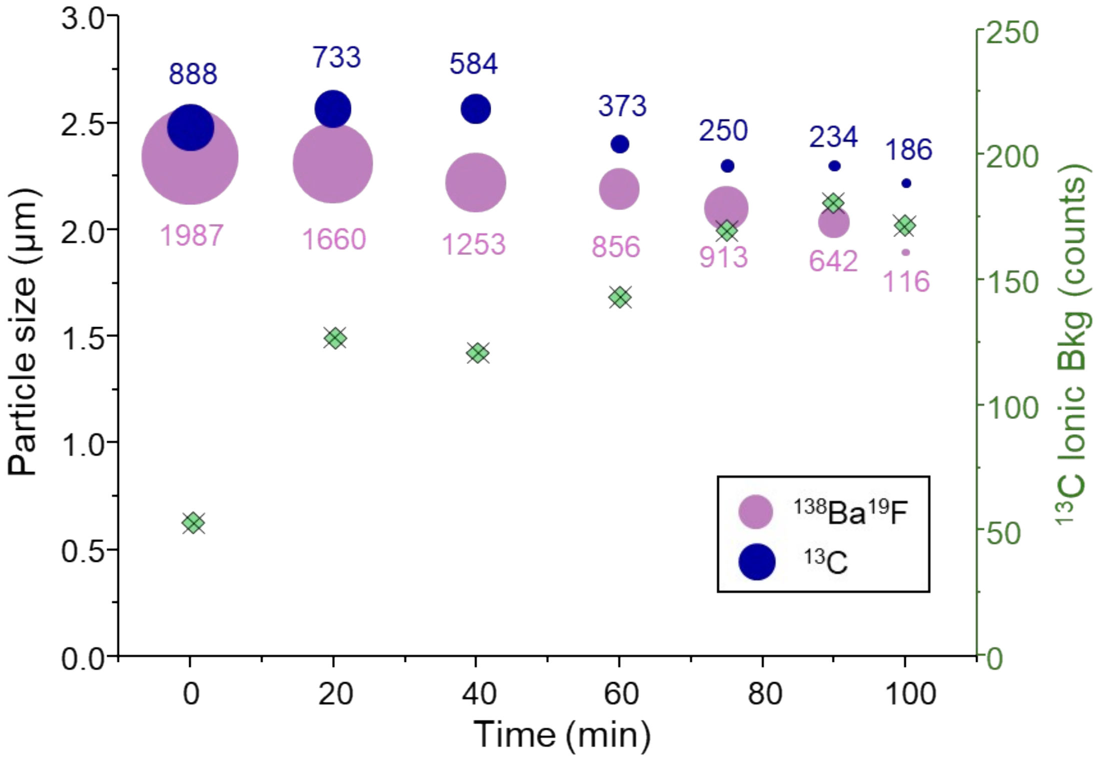

Supplement: JA-040-D5JA00226E-s001 [file JA-040-D5JA00226E-s001.pdf]
